# Internet Use, Cultural Engagement, and Multi-Dimensional Health of Older Adults: A Cross-Sectional Study in China

**DOI:** 10.3389/fpubh.2022.887840

**Published:** 2022-05-27

**Authors:** Wei-chao Chen, Liu Yang, Xiao-yan Wang

**Affiliations:** ^1^School of Journalism and Communication, Hunan Normal University, Changsha, China; ^2^College of Finance and Statistics, Hunan University, Changsha, China

**Keywords:** internet use, multi-dimensional health, influence mechanism, older adults, cultural engagement

## Abstract

With the rapid expansion of the Internet, it continuously penetrates the life of older adults around the world. This study aims to explore the effect of Internet use on the multi-dimensional health of the elderly with the mediating role of cultural engagement. Using data from the Chinese General Social Survey (CGSS) in 2015 and 2017, this study adopts logistic regression and a single-step multiple mediation model to investigate how Internet use affects the multi-dimensional health of older adults in China. The results show that Internet use has positive effects on the self-rated health, physical health, and mental health of the elderly. Endogenous tests, robustness analysis, and sensitivity analysis show that the above conclusions remain robust. Additionally, the mediating effect analysis shows that cultural engagement plays a mediating role in the relationship between Internet use and the three health-related responses. Therefore, to improve the elderly's health level, the government should not only cultivate the ability to use the Internet but also encourage greater cultural engagement amongst the aged.

## Introduction

Aging has become a common global public health concern due to the dramatic increase in the elderly population. As the country with the largest elderly population in the world, China's aging rate has exceeded the world average (1). It is estimated that China's elderly population aged 60 and above will be over 345 million by the end of 2030, posing huge challenges to the supply of medical and public services.

With the popularity of the Internet, it has gradually changed from a way of entertainment to a way of production and life. According to the 48th Statistical Report on the Development of China's Internet (China Internet Network Information Center, 2021), the number of elderly netizens (aged 60 and above) in China reached 123 million in June 2021, accounting for only 12.2% of all netizens (2). At the same time, the existing study found that the Internet continuously penetrates the life of older adults all over the world and increasingly becomes a part of their life, which may have a health effect (3). Previous research has analyzed the direct impact of Internet use on health and obtained many meaningful results. However, the influence mechanism between Internet use and the multi-dimensional health of the elderly is worthy to be further studied.

Cultural engagement is an important indicator to reflect the level of health of the aged. As a core element of successful aging, cultural engagement is one of the essential factors affecting the emotional wellbeing and health of older adults. Many studies have shown that social engagement is negatively associated with some diseases, mortality, and the quality of life of older persons (4). In addition, greater cultural engagement may promote social connectedness and health level among the aged, and further help reduce public health costs. The Internet has an increasingly profound impact on the elderly and gradually changed their lifestyle (5). Studies have confirmed that Internet use can motivate the elderly to participate in entertainment, social, and cultural activities (6), which are the main form of cultural engagement for the elderly.

As such, this study analyzes not only the relationship between Internet use and the multi-dimensional health of elderly people in China but also the effect mechanism among them through the mediating effect analysis using cultural engagement as a mediating variable. It adopts pooled cross-section data from the Chinese General Social Survey (CGSS). Two consecutive data of 2015 and 2017 are used, which are expected to better reflect the impact of Internet use on the multi-dimensional health of the elderly.

## Literature Review

### Effect of Internet Use on the Elderly Health

There are two viewpoints on the effect of Internet use on health. **First, Internet use has a statistically significant negative effect on health**. Some studies found that Internet use may significantly decrease the time spent with friends and local social networking activities, which may increase loneliness and decrease various aspects of the quality of life (7), replacing with weakly connected communication and online entertainment activities (8). Consequently, Internet use further leads to loneliness and social marginalization amongst elderly people (9). In addition, Yang et al. (10) found that Internet use was significantly and negatively associated with the life satisfaction of the Chinese elderly through the way of reducing their perceptions of social justice and communication in real life.

**Second, Internet use has a positive effect on health**. In terms of mental health, Quintana et al. (11) selected life satisfaction, life enjoyment, and self-rated health as proxy indicators. Using the data obtained from the English Longitudinal Study of Aging (ELSA), the empirical analysis results show that Internet use has a significant and positive effect on the mental health of elderly people in the UK. Similarly, Yuan (12) suggested that the more frequently the elderly in Shanghai, China used the Internet, the lower possibility of having psychological problems, especially for the elderly with chronic diseases. Meanwhile, Internet use can relieve older adults' depression and anxiety (13) and the likelihood of dementia and cognitive deterioration (14), and thus promote them to exhibit better health behaviors (15). Also, Internet use could be beneficial for the elderly to obtain more health information, which in turn improved their physical health (16) and mental health (17).

### The Mediating Effect of Cultural Engagement

Being old does not necessarily mean poor physical activity, but it may mean being active and achieving goals in late life (18, 19). According to the study by Nenonen et al. (20), cultural activities were associated with self-rated health and quality of life.

**First**, Internet use may encourage older people to engage in cultural activities more frequently. Compared with non-Internet users, the elderly who used the Internet were not only easier to accept and learn new technologies and lifestyles, but also more optimistic about aging (21), which made them more likely to participate in various cultural activities held both online and offline (22). Further, the elderly could easily obtain relevant information about cultural activities and community events through the Internet, which in turn stimulated them to participate in offline related activities (23). Nasi et al. (24) showed that there was a significantly positive relationship between the frequency of Internet use and cultural activity among the Finnish elderly.

**Second**, as for the relationship between cultural engagement and the elderly's health. According to the Activity Theory, elderly people who like to participate in various activities could be more possible to have a higher level of health (25). Konlaan et al. (26) also found that elderly people who watch movies and participate in art activities frequently have a lower mortality rate. Fancourt and Steptoe (27) found that cultural engagement (e.g., visiting museums/galleries/cinema/theater/concerts) is linked with a lower odds of depression amongst adults. Besides, Wang et al. (28) found that frequent arts participation and cultural attendance play a positive role in alleviating mental distress and promoting levels of life satisfaction, which in turn improve mental health.

## Materials and Methods

### Data Processing

**Figure 1:**
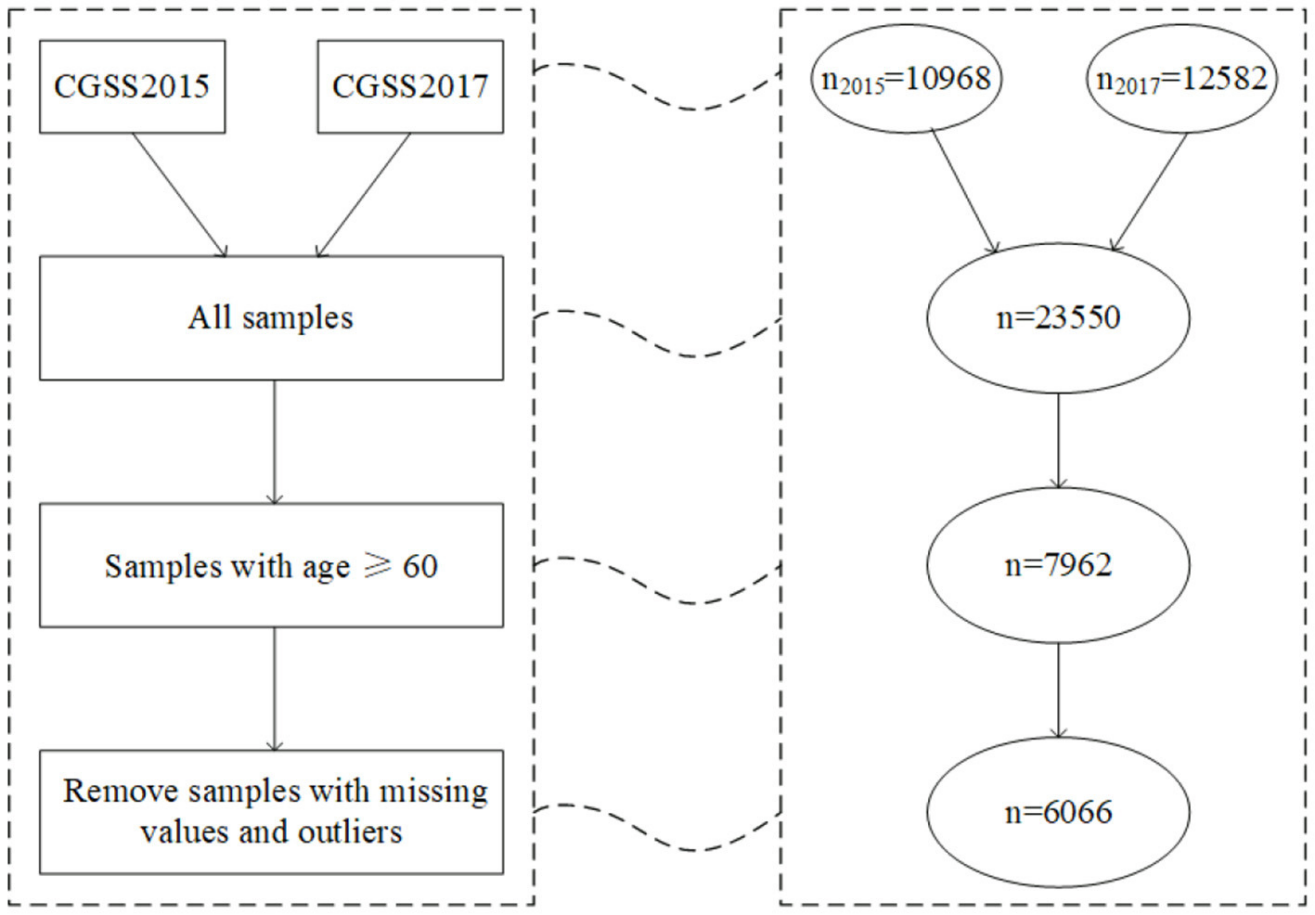
Flowchart of sample selection and preprocessing (*n* represents sample size).

The data used in the study are selected from the Chinese General Social Survey (CGSS) database organized by Renmin University of China. The CGSS adopts multistage stratified sampling to obtain nationally representative samples that span all provinces except for Taiwan, Hong Kong, and Macau. To ensure the data's continuity and improve sample size (hence increasing statistical power), we combine data sets from the two waves in 2015 and 2017 with sample sizes of 10,968 and 12,582, respectively. This data combination is reasonable because the two consecutive data have the same sampling design and questionnaire. Given that the research object is the elderly population over 60 years old, respondents under 60 are excluded from the study. After excluding 1,896 respondents with missing values and outliers, we obtain a final sample with 6,066 respondents. Details about the sample selection and preprocessing are shown in Figure 1.

Empirical analyses in our study are realized by two different statistical software programs: R (a freely available statistical software, version 4.1.0) and Stata (version 16.0). The data were obtained from a publicly accessible database of the Chinese General Social Survey at Renmin University of China Open Research Data platform (http://cgss.ruc.edu.cn/) with a signed data use agreement (29).

### Measurements

#### Dependent Variable

According to Xiong et al. (30) and Zhao and Liu (31), we construct three dependent variables to comprehensively describe health, namely self-rated health, physical health, and mental health. To define self-rated health, we use the question “what do you think of your current state of health,” which has five available answers, namely “1=Very unhealthy,” “2=Less healthy,” “3=Generally,” “4=Healthy,” “5=Very healthy.” We redefine self-rated health=0 for “Very unhealthy,” “Less healthy,” and “Generally,” and otherwise self-rated health=1. Physical health is defined by the question “In the past 4 weeks, how often have health issues affected your work or other daily activities?”. Mental health is measured by the question “In the last 4 weeks, how often have you felt unhappy or depressed?”. The above two questions both have five available answers, including “Never,” “Rarely,” “Sometimes,” “Frequent,” and “Very frequent.” In our analysis, the dependent variable “physical health” and “mental health” are set to 1 (Healthy) if the answer is “Never” or “Rarely,” otherwise set to 0 (Unhealthy).

#### Independent Variable

The independent variable in this study is Internet use, obtained from the question “In the past year, your frequency of Internet use (including mobile Internet) is?”. The available answers are “Never,” “Rarely,” “Sometimes,” “Frequent,” and “Very Frequent.” Referring to the study of Jin and Zhao (32), we redefine “Never” as “non-Internet users” and assign a value of 0, and the other answers are redefined as “Internet users” and assigned a value of 1.

#### Mediating Variable

The mediating variable is cultural engagement. We use the following question in the CGSS: “In the past year, how often have you engaged in the following activities?” (a) “going to the cinema,” (b) “attending cultural events, such as concerts, performances, and exhibitions,” (c) “participate in physical exercise,” and (d) “watching sporting events live.” For each activity (item), the options range from 1 (Never) to 5 (Always). A higher score represents a greater cultural engagement. The above 4 activities are combined to jointly measure the individual level of “cultural engagement.” Therefore, cultural engagement ranges from 4 to 20, with higher scores indicating greater frequency of participation in cultural activities.

#### Control Variable

Previous studies indicated that demographic characteristics and social-psychosocial perception would significantly affect the multi-dimensional health of older adults (33, 34). Considering the effect of other factors on the health of older adults, we also include 6 demographic variables (gender, age, household, education, annual income, and living region) and 3 social-psychosocial perception variables (subjective wellbeing, social trust, and subjective class) as the control variables. The living region (East, Central, and West) is represented by two dummy variables. The descriptive statistical analysis of all variables is shown in Table 1.

**Table 1:** Descriptive statistics of all variables.

**Variable**		**Variables**	**Description of variables**	**Mean**	**Standard deviation**
Dependent variables		Self-rated health	Unhealthy = 0, healthy = 1	0.3968	0.4892
		Physical health		0.5471	0.4978
		Mental health		0.6505	0.4768
Independent variables		Internet use	No use = 0, Use = 1	0.2245	0.4173
Mediating variables		Cultural engagement	A higher score represents a greater cultural engagement.	6.3244	2.4192
	Demographic fctors	Gender	Female = 0, Male = 1	0.4840	0.4997
Control variables (Denoted by *Z*)		Age	Continuous (ranging from 60 to 103)	69.1932	7.2770
		Household	Agricultural household = 0, Non-agricultural household = 1	0.5413	0.4983
		Education	Primary school = 1,Junior high school, Senior high school = 2, College and above = 3	1.5492	0.6378
		Annual income	Annual income (by logarithm)	9.4236	1.3891
	Living region	Eastern	Eastern area = 1, Others = 0	0.4601	0.4984
		Central	Central area = 1, Others = 0	0.3199	0.4665
	Social-psychosocial perception	Subjective wellbeing	Five levels from low to high	3.9606	0.8002
		Social trust	Five levels from low to high	3.6434	0.9301
		Subjective class	Five classes from lower to upper	2.4437	0.8572

### Empirical Model

Since all three dependent variables are binary, logistic regression is adopted to analyze the effect and influence mechanism of Internet use on health. Denote *Z* = (*Z*_1_, ⋯ , *Z*_10_) as all 10 control variables (including dummy variables), *Internet* as Internet use, and *Health*_*k*_ as the *k* th dependent variable (*k* = 1, 2, 3). For each dependent variable, construct the following logistic regression


(1) $$\begin{array}{l} ln\left(p_{ik}|\left(1-p_{ik}\right)\right)=\beta_{0k}+\beta_{1k}Internet_{i}+\overset{10}{\underset{j=1}{\sum }}\gamma_{jk}Z_{ij}+\varepsilon_{ik} \end{array}$$

where *i* = 1, .⋯ , *n* is the *i*th respondent, *p*_*ik*_ = *Prob*(*Health*_*ik*_ = 1) is the probability that the *k*th dependent variable of the *i*th respondent is “health,” ε_*ik*_ is the independently and identically distributed error term. β_*0k*_, β_*1k*_, and γ_*jk*_ are the unknown intercept, the regression coefficient of *Internet*, and regression coefficients of *Z*_*j*_, respectively, for the dependent variable *Health*_*k*_.

It is noted that endogenous problems may exist due to the possible missed important variables. To deal with this problem, we adopt the instrumental variable (IV) method with a two-stage least square (2SLS) estimate. We use the provincial Internet penetration rates as the instrumental variable, which is believed to affect the elderly's Internet use but is irrelevant to health in the existing literature (35, 36). The following 2SLS problem is constructed:


(2) $$\begin{array}{l} Internet_{i}=\varphi_{1}+\phi_{1}IV_{i}+\overset{10}{\underset{j=1}{\sum }}\lambda_{1,jk}Z_{ij}+\varepsilon_{i0} \end{array}$$

(3) $$\begin{array}{l} Health_{ik}=\varphi_{2k}+\phi_{2k}\bar{Internet_{i}}+\overset{10}{\underset{j=1}{\sum }}\lambda_{2,jk}Z_{ij}+\varepsilon_{ik} \end{array}$$

where *IV*_*i*_ is the instrumental variable, and $\bar{Internet_{i}}$ is the predicted value of *Internet*_*i*_ from (2). *Internet*_*i*_, *Z*_*ij*_, and ε are the same as above. φ, ϕ, and λ are parameters to be estimated.

Meanwhile, we also conduct robustness analysis by adopting the propensity score matching (PSM) method. Based on the independent variable Internet use, the samples can be divided into the treatment group (Internet users) and the control group (non-Internet users). We then identify control variables as many as possible that affect both the dependent variable health and the independent variable Internet use. The treatment effect of Internet use on the health of older adults is as follows:

(4) $$\begin{array}{l} \begin{array}{c} Prob(D_{i}=1|Z_{i})=\frac{e^{\alpha_{0}+\sum_{j=1}^{10}\alpha_{j}Z_{ij}}}{1+e^{\alpha_{0}+\sum_{j=1}^{10}\alpha_{j}Z_{ij}}} \end{array} \end{array}$$

In Equation (4), *Z*_*i*_ is the control variable of the *i*th older adult. *D*_*i*_ is the indicator variable. *D*_*i*_= 1 indicates that the *i* th older adult has internet access, and *D*_*i*_= 0 indicates that older adults *i* do not have internet access.

To further obtain robust matching results, this study used three common matching algorithms, i.e., K-nearest neighbor matching, radius matching, kernel matching, and caliper nearest neighbor matching. The average treatment effect on the treated (ATT) for multi-dimensional health is given as


(5) $$\begin{array}{l} \begin{array}{c} \begin{array}{l} ATT_{k}=E(Health_{ik}^{T}-Health_{ik}^{C}|D_{i}=1)\;\\ \;=E(Health_{ik}^{T}|D_{i}=1)-E(Health_{ik}^{C}|D_{i}=1) \end{array} \end{array} \end{array}$$

In Equation (5), $Health_{ik\;}^{T}$and $Health_{ik}^{C}$ represent the observed and unobserved *Health*_*k*_ of the treatment groups, respectively.

## Results

### Benchmark Regression

Before empirical analysis, we conduct a multicollinearity test. The VIF (variance inflation factor) is far below the critical value of 10 with a mean of 1.42 and a maximum of 2.13. Therefore, multicollinearity does not exist in our data.

To explore the effect of the internet use on the health, we adopt the logistic regression and denote Model_*i*_, *i* = 1, 2, 3 as the model for dependent variables “Self-rated health,” “Physical health,” and “Mental health,” respectively. Estimation results are shown in Table 2.

**Table 2:** Logistic regression estimation results of Internet use on multi-dimensional health.

**Variable**	**Model 1**	**Model 2**	**Model 3**
	**Self-rated health**	**Physical health**	**Mental health**
Internet use	0.1552^**^ (0.075)	0.3250^***^ (0.079)	0.2033^**^ (0.084)
Gender	−0.3478^***^ (0.056)	−0.3054^***^ (0.057)	−0.2425^***^ (0.059)
Age	−0.0228^***^ (0.003)	−0.0323^***^ (0.004)	−0.0052 (0.004)
Household	−0.1293^*^ (0.079)	0.1975^**^ (0.078)	0.0551 (0.082)
Education	−0.0574 (0.053)	−0.0289 (0.055)	0.0551 (0.059)
Annual income	0.1560^***^ (0.029)	0.2251^***^ (0.029)	0.1713^***^ (0.029)
East	0.1550^**^ (0.078)	0.4864^***^ (0.078)	0.4816^***^ (0.080)
Central	0.2008^***^ (0.077)	0.2494^***^ (0.075)	0.3487^***^ (0.076)
Subjective wellbeing	0.5072^***^ (0.040)	0.4292^***^ (0.038)	0.6803^***^ (0.039)
Social trust	0.0879^***^ (0.030)	0.1358^***^ (0.030)	0.0918^***^ (0.031)
Subjective class	0.2032^***^ (0.034)	0.1864^***^ (0.034)	0.1818^***^ (0.036)
*N*	6,066	6,066	6,066
Chi-square (*p*-value)	5.165(*p* = 0.739)	6.708 (*p* = 0.568)	5.504 (*p* = 0.702)

* *p < 0.1*,

** *p < 0.05*,

*** *p < 0.01 (Standard errors are in parentheses)*.

From Table 2, we can observe that almost all variables have significant effects on the health. Internet use has a positive effect on all three dependent variables. Taking a look at the coefficients of the control variables, compared with men, women's self-rated, physical, and mental health levels are higher. With the increase of age, the levels of self-rated health and physical health will decline, but age has no significant effect on mental health. The physical health of agricultural households is higher than that of groups with non-agricultural household. Annual income has a positive effect on the health level of all three dimensions. From the perspective of geographical location, the people in the central and eastern regions have a high probability to be healthy (in terms of all three types of health) than people in the western area. In terms of socio-economic factors, subjective wellbeing, social trust, and subjective class show significant positive effects on all the three health-related dependent variables.

The goodness-of-fit tests based on Hosmer-Lemeshow Chisq-square statistics in Table 2 show that the constructed models fit the data well, which may verify that the variables we choose for model construction are appropriate.

### Endogenous Treatment: Instrumental Variables Approach

There may be an endogeneity problem between Internet use and the health of older adults. Therefore, the 2SLS method is used to reduce the bias caused by the endogeneity problem. 2SLS method is usually used to analyze it and the validity of the instrumental variables (37). Theoretically, effective instrumental variables must be uncorrelated with random error. Meanwhile, they must be highly correlated with endogenous variables. Here, we choose the provincial Internet penetration rates as the IV. On the one hand, Internet usage is related to the provincial Internet penetration rates. On the other hand, the Internet penetration rate is not related to the elderly's health at the micro-individual level.

To conduct 2SLS method, first, we estimate the effect of provincial Internet penetration rates on Internet use. Second, we estimate the effect of Internet use on health by regression. The estimation results are reported in Table 3. For a single instrumental variable, F-statistics under 10 are thought to suggest a problem of weak instruments (38). In this study, the F-statistic implied by this first-stage regression is 213.28, which allays any concerns about weak instruments.

**Table 3:** The treatment of endogeneity: instrumental variable model.

**Variables**	**Results of the first-stage regression**	**Results of the second-stage** **regression**		
		**Self-rated** **health**	**Physical** **health**	**Mental** **health**
Instrumental variable (provincial Internet penetration rates)	0.0073^***^ (0.001)			
Internet use		0.8832^***^ (0.174)	0.1462^***^ (0.141)	0.2918^*^ (0.140)
Control variables	Yes	Yes	Yes	Yes
Sample size	6,066	6,066	6,066	6,066
*adj*.*R*^2^	0.2780			
F value of the first-stage regression	213.28 (*p* < 0.0001)			

* *p < 0.1*,

^**^p < 0.05,

*** *p < 0.01*.

The regression results of the full sample show that the regression coefficient of the provincial Internet penetration rates on the health of the elderly is 0.0073, which is significant at the 1% level. Next, the coefficients of Internet use in the second-stage regression on the self-rated, physical, and mental health of the elderly are 0.8832, 0.1462, and 0.2918, respectively. The results show that Internet use has a significant positive effect on all dimensions of health. As a result, the relationship between Internet use and the multi-dimensional health of older adults has been further verified.

### Robustness Analysis

To obtain the net effect of Internet use on the self-rated health of the elderly, propensity score matching (PSM) is selected to test the robustness of logistic regression results. We divide the sample into two groups: the treatment group (using the Internet) and the control group (not using the Internet). Before adopting PSM, the sample must pass the balance test, which ensures that no systematic difference exists between the treatment group and the control group after matching except for the key explanatory variables. We employ the PSM method to generate a matched comparison group for our analysis. The results of the balance tests are presented in Table 4.

**Table 4:** Results of balance test.

**Variable**	**Mean**				***t*** **-test**	
	**Sample**	**Treatment**	**Control**	**Deviation rate**	***t*-value**	**p>|t|**
		**group**	**group**	**(%)**		
Gender	U	0.448	0.494	−9.3	−3.03	0.002
	M	0.453	0.459	−1.2	−0.32	0.748
Age	U	66.889	69.860	−43.6	−13.47	0.000
	M	67.006	66.958	0.7	0.20	0.843
Household	U	0.872	0.446	100.5	29.71	0.000
	M	0.869	0.864	1.2	0.38	0.705
Education	U	2.071	1.398	114.0	38.19	0.000
	M	2.051	2.060	−1.5	−0.38	0.707
Annual income	U	10.417	9.136	110.5	32.48	0.000
	M	10.393	10.371	1.9	0.61	0.542
Subjective wellbeing	U	4.021	3.943	10.2	3.18	0.001
	M	4.019	3.996	3.1	0.80	0.425
Social trust	U	3.541	3.673	−13.9	−4.62	0.000
	M	3.542	3.513	3.1	0.77	0.443
Subjective class	U	2.600	2.399	23.8	7.67	0.000
	M	2.586	2.588	−0.2	−0.06	0.949

*The results in the table are obtained by using the radius matching method*.

Taking the balance test results of radius matching as an example, the standardized deviation of all variables is controlled within the desired 5% after matching. All of the *t*-values are not significant after matching, which shows that the difference between the treatment and control groups is not significant after applying the PSM. The results show that the method of PSM is similar to the results of random experiments, indicating a better matching effect. At this time, the results of the balance test and the effect of average Internet Internet use on physical and mental health are presented in Supplementary Tables 1–4. In conclusion, the results are consistent with the aforementioned logistic regression results. Specifically, Internet use is significantly and positively associated with self-rated, physical, and mental health among older adults both before and after matching.

This paper firstly estimates ATT before matching, and the results are shown in Table 5. The average treatment effect before matching is significantly higher than that after matching, which means that if the selection bias is not considered, the influence of the Internet on self-rated health will be overestimated. There are four different matching methods adopted to calculate the average treatment effect. The estimation results of different matching methods are similar, indicating that this study is not sensitive to matching methods and has good robustness.

**Table 5:** The average treatment effect of Internet use on self-rated health.

**Matching Method**	**Treatment group (1)**	**Control group (2)**	**ATT value (1)-(2)**	**Standard deviation**	***t*-value**
Before the match ATT	0.470	0.375	0.095	0.015	6.34^***^
After the match ATT					
K-nearest neighbor matching (k = 4)	0.470	0.428	0.042	0.023	1.83^*^
Radius matching method	0.467	0.423	0.043	0.021	2.06^**^
Kernel matching	0.470	0.375	0.095	0.015	2.31^**^
Caliper nearest neighbor matching	0.468	0.427	0.041	0.022	1.80^*^

* *p < 0.1*,

** *p < 0.05*,

*** *p < 0.01*.

### Sensitivity Analysis

This study applies sensitivity analysis to test the robustness of the result. Rosenbaum's approach in particular focuses on the hidden biases that can change the results of the treatment effectiveness obtained. We use the command “rbounds” in STATA to conduct the “Rosenbaum bounds” analysis. Generally, the higher the value of Γ, the lower the hidden bias would be. Generally, if the existing conclusion becomes insignificant when the value of Γ (gamma) is very large (usually close to 2), it can be considered that the conclusion is tenable (39).

The results of the sensitivity analysis are displayed in Supplementary Tables 5–7. The critical values of gamma (Γ) range from 1 to 2. For self-rated health, when Γ increases to 1.8, we can observe a significant sensitivity (Supplementary Table 5). For physical health, with Γ = 1.5 a significant sensitivity at the 10% significance level is observed (Supplementary Table 6). For mental health, with Γ = 1.4 a significant sensitivity at the 10% significance level is observed (Supplementary Table 7). The above three Γ values are closed to the threshold value 2. This can partly support that the PSM analysis results are reliable.

### Heterogeneity Analysis

We further examine the differences in the effect of Internet use on the health of seniors among different age groups and household groups. As shown in Table 6, regarding age differences, Internet use has a significant positive effect on the self-rated, physical, and mental health of the elderly aged 60–69, however, it has no significant effect on the health of the group over 70 years old.

**Table 6:** Regression results by household.

**Variables**	**Age: 60–69**			**Age** **≥** **70**		
	**Self-rated health**	**Physical health**	**Mental health**	**Self-rated health**	**Physical health**	**Mental health**
Internet use	0.1707^*^ (0.090)	0.3620^***^ (0.096)	0.2113^**^ (0.101)	0.1204 (0.139)	0.1994 (0.140)	0.1356 (0.157)
Control variables	Yes	Yes	Yes	Yes	Yes	Yes
Chi-square /*p*-value	3.435 (*p* = 0.904)	9.570 (*p* = 0.296)	9.283 (*p* = 0.318)	5.033 (*p* = 0.754)	7.100 (*p* = 0.525)	12.329 (*p* = 0.137)
*N*	3,859			2,207		

* *p < 0.1*,

** *p < 0.05*,

*** *p < 0.01*.

From the perspective of the household, Internet use has a significant positive effect on the self-rated health of the agricultural household elderly, but not so much for the non-agricultural households. However, the effects of Internet use on physical health and mental health of non-agricultural older adults is significantly greater than that of the group with agricultural household (Table 7).

**Table 7:** Regression results by household.

**Variables**	**Agricultural household**			**Non-agricultural household**		
	**Self-rated health**	**Physical health**	**Mental health**	**Self-rated health**	**Physical health**	**Mental health**
Internet use	0.3501^**^ (0.171)	0.3762^**^ (0.175)	0.1720 (0.179)	0.1408^*^ (0.085)	0.2878^***^ (0.090)	0.1758^*^ (0.098)
Control variables	Yes	Yes	Yes	Yes	Yes	Yes
Chi-square /*p*-value	0.765 (*p* = 0.999)	10.685 (*p* = 0.220)	2.716 (*p* = 0.950)	3.154 (*p* = 0.924)	10.965 (*p* = 0.203)	19.366 (*p* = 0.130)
*N*	2,782			3,284		

* *p < 0.1*,

** *p < 0.05*,

*** *p < 0.01*.

### Mechanism Analysis

The results of the logistic regression discussed above also suggest a significant positive effect of Internet use on the multi-dimensional health of older adults. Here, we further explored whether cultural engagement is an intermediary factor driving this relationship. The single-step multiple mediation analysis introduced by Hayes (40) with bootstrapping using 2,000 bootstrap samples and 95% Bias Corrected (BC) bootstrap confidence interval (CI) is used in this study.

Referring to the study by Cheung and Lau (41), the effect is considered to be significant if zero is not included in the confidence intervals. As shown in Table 8, first, the effects of Internet use on self-rated health, physical health, and mental health are all significant and positive, with a standardized estimate of 0.014 [95% CI = (0.007, 0.024)], 0.022 [95% CI= (0.015, 0.032)], and 0.011 [95% CI= (0.004, 0.019)], respectively. An indirect effect is declared significant for the given sample if the confidence interval excludes zero (42). Hence, the indirect effect of Internet use on self-rated health, physical health, and mental health is mediated through cultural engagement.

**Table 8:** The mediating effect of cultural engagement.

**Variables**	**Effect**	**Estimate**	**SE**	**95% CI (Confidence interval)**	
				**LLCI**	**ULCI**
Self-rated health	Mediating effect	0.016	0.004	0.009	0.023
	Direct effect	0.021	0.017	−0.013	0.055
	Total effect	0.037	0.017	0.004	0.071
Physical health	Mediating effect	0.020	0.003	0.014	0.027
	Direct effect	0.047	0.017	0.017	0.082
	Total effect	0.067	0.017	0.036	0.100
Mental health	Mediating effect	0.008	0.003	0.002	0.014
	Direct effect	0.028	0.015	−0.002	0.060
	Total effect	0.036	0.015	0.008	0.068

## Discussion

To better explore the relationship between Internet use and multi-dimensional health among Chinese older adults, this study adopts a comprehensive empirical analysis using the CGSS data in 2015 and 2017. Logistic regression is used to obtain how Internet use affects the three types of health of the elderly. To verify the analysis, 2SLS method and PSM method are adopted for endogenous and robustness tests, respectively. Moreover, this paper also conducts a heterogeneity analysis for different age groups and household groups. To explore the mechanism of the effect of Internet use on health, we use cultural engagement as a mediating variable for mediating effect analysis. The results are as follows:

First, Internet use has a positive effect on self-rated health, which is similar to the previous study (43). Internet use significantly and positively effects physical health, which is also consistent with previous findings (44). Also, Internet use is significantly positively associated with mental health, which is consistent with prior work of Keane et al. (45) and Chang and Im (46). In other words, the more frequently older persons use the Internet, the greater the impact on self-rated health, physical health, and mental health will be. This possible reason may be that the information and resources on the Internet are more abundant, which helps seniors enrich their lives and keep connected to their social networks.

Second, the heterogeneity analysis shows that for the group aged from 60 to 69, using the Internet may improve their levels of self-rated, physical, and mental health, whereas, for the elderly over the age of 70, Internet use has not yet shown significant effect on their health level. The results are consistent with the study of Yang and He (47). The possible reason for the above findings is that the younger older adults have fewer obstacles to Internet use, so they can obtain various types of information and resources on the Internet more conveniently. Additionally, for the elderly with agricultural household, the increasing self-rated health effect is more apparent when using the Internet. Perhaps this is because the Internet is still a novelty for the elderly with agricultural household. However, the physical and mental health of the aged with agricultural household is significantly lower than their counterparts in the non-agricultural group. The possible reason is that non-agricultural household seniors benefit not just from better quality network access, but also from a high level of Internet skills for social and leisure participation.

Third, our findings demonstrate that cultural engagement is introduced as a mediating variable to identify the influence mechanism of Internet use on three dimensions of health. On the one hand, Internet use will promote cultural engagement activities, and this finding is consistent with the study of Cilesiz (48). Internet use can effectively reduce the cost of information acquisition, entertainment, and consumption among the elderly. Therefore, older adults may use the Internet as a medium to get information about cultural activities, which could further encourage greater cultural engagement of the elderly (49). Moreover, older people who use the Internet are more likely to accept new technologies and lifestyles, making them more likely to participate in a variety of cultural activities. On the other hand, a significant positive correlation is found between cultural engagement activities and the level of self-rated health, physical health, and mental health among the elderly. This is evidenced in previous studies (29, 50).

Based on the above empirical analysis results, this paper puts forward some suggestions as follows: (1) Improve older adults' attitudes toward Internet use. Given that Internet technology is thought to be more challenging for the elderly to learn (51), they fail to accept the convenience brought by the Internet. In this regard, a targeted training service to enhance the ability of older adults to use the Internet will help them become familiar with the various applications (e.g., health management, leisure, and social contact), which in turn bridge the digital divide and achieve active aging. Meanwhile, relevant departments should cultivate the elderly's ability how to distinguish quality health information from inaccurate, misleading, or fraudulent material. (2) The government should help to create an elderly-friendly environment by building activity centers and increasing public service fiscal expenditures. Furthermore, social resources should be mobilized for investment in the elderly-care industry. (3) The local community should organize various forms of cultural activities among the aged and widely publicize them through the Internet, to stimulate the interest of the elderly in cultural participation.

This study enriches and broadens previous research and contributes to the literature as follows. Previous research has used data from only 1 year to explore the impact of internet use on a single dimension of health. In comparison with existing studies, we use the CGSS data from 2015 to 2017 to ensure the data's availability and continuity. In addition, we used an innovative research perspective, the study divides health into three dimensions (self-rated, physical, and mental health), providing a multi-dimensional consideration of health connotation. More importantly, our findings provide evidence for the first time that cultural engagement acts as an important mediating variable between Internet use and the health of older adults. It not only provides empirical support for improving the health status of older adults but also supports the promotion of policies on active aging.

However, this paper also has some limitations. Due to the availability of variables of secondary data, only cultural engagement is used as a mediating variable. Further, given the absence of broader measures of economic activities, family activities, and political activities, it is difficult to accurately reflect the mediating effect of active aging between Internet use and the health of older adults. However, these deficiencies will provide research directions for further research in the future. These limitations are worthy of more relevant future studies.

## Conclusion

Based on a national sample of Chinese older adults, this study establishes a new conceptual framework to explain the mechanism of how Internet use affects the multi-dimensional health of older adults under the mediating role of cultural engagement. This study unveils that Internet use directly affected health among older adults, and cultural engagement plays an intermediary role between Internet use and multi-dimensional health.

## Data Availability Statement

Publicly available datasets were analyzed in this study. This data can be found at: http://cgss.ruc.edu.cn/.

## Author Contributions

W-cC: conceptualized the paper and designed the methodology. W-cC and X-yW: data analysis. W-cC and LY: writing—original draft preparation. W-cC, LY, and X-yW: writing—review and editing. All authors have read and agreed to the published version of the manuscript.

## Funding

This research was funded by the National Social Science Fund of China (20AXW010), the Philosophy and Social Science Foundation of Hunan Province (18YBQ114), Natural Science Foundation of Changsha City (kq2202180), and Evaluation Committee of Social Science Achievements of Hunan Province (XSP22YBZ003).

## Conflict of Interest

The authors declare that the research was conducted in the absence of any commercial or financial relationships that could be construed as a potential conflict of interest.

## Publisher's Note

All claims expressed in this article are solely those of the authors and do not necessarily represent those of their affiliated organizations, or those of the publisher, the editors and the reviewers. Any product that may be evaluated in this article, or claim that may be made by its manufacturer, is not guaranteed or endorsed by the publisher.
