# Supplementary material for: Internet Use, Cultural Engagement, and Multi-Dimensional Health of Older Adults: A Cross-Sectional Study in China
Source: Front Public Health. 2022 May 27;10:887840. doi: 10.3389/fpubh.2022.887840 (PMC9185139; doi:10.3389/fpubh.2022.887840)
Supplement: Supplementary file 1 [file Data_Sheet_1.pdf]

The results of the balance test and the effect of average Internet Internet use on physical health are presented in supplemental documents, Table S1-S4.

TABLE S 1 | Results of balance test.

Variable	Mean				t-test	
	Sample	Treatment group	Control group	Deviation rate (%)	t-value	p>|t|
Gender	U	0.448	0.494	-9.3	-3.03	0.002
	M	0.448	0.460	-2.4	-0.64	0.524
Age	U	66.889	69.860	-43.6	-13.470	0.000
	M	66.892	66.865	0.4	0.11	0.909
Household	U	0.872	0.446	100.5	29.710	0.000
	M	0.872	0.869	0.7	0.21	0.830
Education	U	2.071	1.398	114.0	38.190	0.000
	M	2.070	2.090	-3.4	-0.84	0.399
Annual income	U	10.417	9.136	110.5	32.480	0.000
	M	10.417	10.366	4.4	1.44	0.150
Subjective well-being	U	4.021	3.943	10.2	3.180	0.001
	M	4.021	3.995	3.5	0.92	0.359
Social trust	U	3.541	3.673	-13.9	-4.620	0.000
	M	3.541	3.482	6.2	1.53	0.125
Subjective class	U	2.600	2.399	23.8	7.670	0.000
	M	2.598	2.582	2.0	0.53	0.559

The results in the table were obtained by using the K-nearest neighbor matching（k=4） method.

TABLE S 2 | The average treatment effect of Internet use on physical health.

Matching Method	Treatment group (1)	Control group (2)	ATT value (1)－(2)	Standard deviation	t-value
Before the match ATT	0.707	0.501	0.206	0.015	13.67***
After the match ATT					
K-nearest neighbor matching（k=4）	0.708	0.630	0.078	0.023	3.47***
Radius matching method	0.705	0.625	0.079	0.020	3.81***
Kernel matching	0.707	0.632	0.074	0.020	3.61***
Mahalanobis matching	0.707	0.628	0.078	0.020	3.81***

*"***", "**", and "*" indicate significance at 1%, 5%, and 10% levels, respectively.*

The results of the balance test and the effect of average Internet Internet use on mental health are presented in supplemental documents, Table S1-S4.

TABLE S 3 | Results of balance test.

Variable	Mean				t-test	
	Sample	Treatment group	Control group	Deviation rate (%)	t-value	p>|t|
Gender	U	0.448	0.494	-9.3	-3.03	0.002
	M	0.453	0.459	-1.2	-0.32	0.748
Age	U	66.889	69.860	-43.6	-13.47	0.000
	M	67.006	66.958	0.7	0.20	0.843
Household	U	0.872	0.446	100.5	29.71	0.000
	M	0.869	0.864	1.2	-0.38	0.705
Education	U	2.071	1.398	114.0	38.19	0.000
	M	2.051	2.060	-1.5	0.38	0.707
Annual income	U	10.417	9.136	110.5	32.48	0.000
	M	10.393	10.371	1.9	0.61	0.542
Subjective well-being	U	4.021	3.943	10.2	3.18	0.001
	M	4.019	3.996	3.1	0.80	0.425
Social trust	U	3.541	3.673	-13.9	-4.62	0.000
	M	3.542	3.513	3.1	0.77	0.443
Subjective class	U	2.600	2.399	23.8	7.67	0.000
	M	2.586	2.588	-0.2	-0.06	0.949

The results in the table were obtained by using the K-nearest neighbor matching（k=4） method.

TABLE S 4 | The average treatment effect of Internet use on mental health.

Matching Method	Treatment group (1)	Control group (2)	ATT value (1)－(2)	Standard deviation	t-value
Before the match ATT	0.755	0.620	0.134	0.015	9.23***
After the match ATT					
Mahalanobis matching	0.754	0.717	0.037	0.019	1.91*
Radius matching method	0.753	0.718	0.034	0.020	1.71*
Kernel matching	0.754	0.717	0.036	0.019	1.84*
Caliper nearest neighbor matching	0.752	0.717	0.034	0.021	1.64*

*"***", "**", and "*" indicate significance at 1%, 5%, and 10% levels, respectively.*

TABLE S 5 | Sensitivity analysis (self-rated health as the dependent variable).

	Significance levels		Hodges-Lehmann point estimates		95% Confidence intervals	
Gamma (Γ)	Minimum	Maximum	Minimum	Maximum	Minimum	Maximum
1	0	0	0.071	0.071	0.061	0.079
1.1	0	0	0.064	0.077	0.053	0.085
1.2	< 0.0001	0	0.057	0.083	0.046	0.092
1.3	< 0.0001	0	0.050	0.089	0.039	0.097
1.4	< 0.0001	0	0.044	0.094	0.031	0.103
1.5	< 0.0001	0	0.037	0.098	0.023	0.108
1.6	0.001	0	0.030	0.103	0.014	0.113
1.7	0.016	0	0.023	0.107	0.002	0.119
1.8	0.098	0	0.015	0.112	-0.011	0.125
1.9	0.315	0	0.006	0.117	-0.032	0.130
2.0	0.312	0	-0.004	0.122	-0.064	0.135

The results in the table are obtained by using the radius matching method.

TABLE S 6 | Sensitivity analysis (physical health as the dependent variable).

	Significance levels		Hodges-Lehmann point estimates		95% Confidence intervals	
Gamma (Γ)	Minimum	Maximum	Minimum	Maximum	Minimum	Maximum
1	< 0.0001	0	0.125	0.125	0.125	0.015
1.1	< 0.0001	0	0.125	0.150	0.075	0.200
1.2	< 0.0001	0	0.100	0.187	0.025	0.236
1.3	0.001	0	0.042	0.225	0.001	0.250
1.4	0.031	0	< 0.0001	0.250	< -0.0001	0.250
1.5	0.215	0	< -0.0001	0.250	< -0.0001	0.250

The results in the table are obtained by using the radius matching method.

TABLE S 7 | Sensitivity analysis (mental health as the dependent variable).

	Significance levels		Hodges-Lehmann point estimates		95% Confidence intervals	
Gamma (Γ)	Minimum	Maximum	Minimum	Maximum	Minimum	Maximum
1	< 0.0001	0	0.1250	0.125	0.100	0.142
1.1	< 0.0001	0	0.100	0.141	0.083	0.166
1.2	< 0.0001	0	0.083	0.156	0.036	0.183
1.3	0.005	0	0.050	0.174	-0.0001	0.200
1.4	0.082	0	0.0113	0.193	-0.0001	0.214
1.5	0.373	0	-0.0001	0.200	-0.05	0.225

The results in the table are obtained by using the radius matching method.
